# Dopaminergic basis of the psychosis-prone personality investigated with functional magnetic resonance imaging of procedural learning

**DOI:** 10.3389/fnhum.2013.00130

**Published:** 2013-04-15

**Authors:** Ulrich Ettinger, Philip J. Corr, Ardeshier Mofidi, Steven C. R. Williams, Veena Kumari

**Affiliations:** ^1^Department of Psychology, University of BonnBonn, Germany; ^2^Department of Neuroimaging, Institute of Psychiatry, King's College LondonLondon, UK; ^3^Department of Psychology, City UniversityLondon, UK; ^4^Department of Psychology, Institute of Psychiatry, King's College LondonLondon, UK; ^5^NIHR Biomedical Research Centre for Mental Health, The Institute of Psychiatry, South London and Maudsley NHS TrustLondon, UK

**Keywords:** schizotypy, sequence learning, dopamine, striatum, thalamus

## Abstract

Previous evidence shows a reliable association between psychosis-prone (especially schizotypal) personality traits and performance on dopamine (DA)-sensitive tasks (e.g., prepulse inhibition and antisaccade). Here, we used blood oxygen level-dependent (BOLD) fMRI and an established procedural learning (PL) task to examine the dopaminergic basis of two aspects of psychosis-proneness (specific schizotypy and general psychoticism). Thirty healthy participants (final *N* = 26) underwent fMRI during a blocked, periodic sequence-learning task which, in previous studies, has been shown to reveal impaired performance in schizophrenia patients given drugs blocking the DA D2 receptor subtype (DRD2), and to correspond with manipulation of DA activity and elicit fronto-striatal-cerebellar activity in healthy people. Psychosis-proneness was indexed by the Psychoticism (P) scale of the Eysenck Personality Questionnaire-Revised (EPQ-R; 1991) and the Schizotypal Personality Scale (STA; 1984). EPQ-R Extraversion and Neuroticism scores were also examined to establish discriminant validity. We found a positive correlation between the two psychosis-proneness measures (*r* = 0.43), and a robust and unique positive association between EPQ-R P and BOLD signal in the putamen, caudate, thalamus, insula, and frontal regions. STA schizotypy score correlated positively with activity in the right middle temporal gyrus. As DA is a key transmitter in the basal ganglia, and the thalamus contains the highest levels of DRD2 receptors of all extrastriatal regions, our results support a dopaminergic basis of psychosis-proneness as measured by the EPQ-R Psychoticism.

## Introduction

The psychosis-prone personality has been measured in a number of different ways. One approach has been to measure clinically-informed specific traits, as seen in various schizotypy questionnaires (Claridge and Broks, 1984). The second approach has been to measure a more general construct as developed, principally, by psychoticism (P; Eysenck, 1992). Sometimes, these constructs are combined into a single measurement instrument (e.g., Claridge et al., 1996). Debate continues concerning the precise roles played by these different factors in the psychosis-prone personality.

Most work has focused on the construct of schizotypy (Rado, 1953) which is a trait placed within, or in close proximity to, the schizophrenia spectrum (e.g., Meehl, 1990; Claridge, 1997; Gruzelier, 2002; Raine, 2006). It overlaps with schizophrenia at various levels of measurement, including the descriptive clinical level (Lenzenweger, 2010), the cognitive level (e.g., Cochrane et al., 2012; for review, see Giakoumaki, 2012) and the level of neurobiology (e.g., Bollini et al., 2007; Aichert et al., 2012). The enduring hypotheses of, and consistent evidence for, abnormal dopamine (DA) function in schizophrenia (Gray et al., 1991; Di Forti et al., 2007; Howes and Kapur, 2009; Eyles et al., 2012), combined with continuum models of psychosis (Johns and van Os, 2001), suggest that schizotypal personality, or at least some dimensions of it, should also have a dopaminergic basis.

Pharmacological studies provide direct evidence of an association between schizotypy and alterations in DA neurotransmission (Mohr et al., 2005; Woodward et al., 2011; Koychev et al., 2012). Several studies have also shown schizophrenia-like performance on DA-sensitive tasks, for example reduced latent inhibition (review, Kumari and Ettinger, 2009; Granger et al., 2012), reduced prepulse inhibition (Evans et al., 2005; Kumari et al., 2005), increased antisaccade errors (O'Driscoll et al., 1998; Ettinger et al., 2005; Gooding et al., 2006), reduced Kamin blocking effect (Moran et al., 2003), aberrant salience related to dysfunctional reward learning (Roiser et al., 2009) and altered salience attribution (Galdos et al., 2011) in association with various dimensions of (high) schizotypy. Further support comes from functional imaging, for example, in showing a negative relationship between psychometric schizotypy and activity in the striatum and thalamus during antisaccade task (Aichert et al., 2012), and a positive relationship between fronto-striatal prediction error signal and delusion-like beliefs in healthy people (Corlett and Fletcher, 2012), compatible with what has been found in schizophrenia (Raemaekers et al., 2002; Corlett et al., 2007).

Much less work has been conducted using the more general trait of psychoticism, but where such studies exist there is evidence of a DA basis. For example, individuals scoring high on psychoticism show reduced latent inhibition (Kumari and Ettinger, 2009); they also show lower prepulse inhibition (Kumari et al., 1997, 2008a) and less striatal-thalamic activity during prepulse inhibition (Kumari et al., 2008a) in line with what has been found in schizophrenia (Kumari et al., 2003, 2007). There are negative correlations between psychoticism and cerebral perfusion in the basal ganglia (putamen and caudate) and thalamus (O'Gorman et al., 2006)—cerebral perfusion is a fundamental physiological quantity reflecting the rate of delivery of oxygen and other nutrients to an organ or tissue. Psychoticism has also been associated with decreased metabolic rate in the basal ganglia and thalamus (Haier et al., 1987). The psychoticism–DA relationship is consistent with the negative association between psychoticism and DA D2 binding (Gray et al., 1994) and resting fMRI signal in the basal ganglia and thalamus (Kumari et al., 2004). In relation to the experimental manipulation of DA, Corr and Kumari (2000) reported an interaction of psychoticism with (5 and 10 mg) d-amphetamine challenge on self-reported mood.

The motivation for the present study was to compare the validity of these two forms of psychosis-proneness personality constructs in relation to the functional neuroanatomical basis of a strongly DA-sensitive procedural learning (PL) task [a variant of the serial reaction time task (SRT) which involves learning of sequences]. PL is a type of rule-based learning in which performance facilitation occurs with practice on task without the need for conscious awareness (Cohen and Squire, 1980; Squire and Zola-Morgan, 1988). PL is generally independent of intelligence and performance on tests of declarative learning and memory (Feldman et al., 1995). PL is sensitive to changes in the DA system (Foerde and Shohamy, 2011), with most prominent effects seen in the dorsal striatum (improved by moderately elevated DA levels and impaired by decreased DA levels), and there is no clear evidence so far for its sensitivity to serotonergic, noradrenergic, and cholinergic systems (Uddén et al., 2010). The performance on the PL task we used in this study has been shown previously to improve and worsen in healthy people following the acute administration of a DA-agonist, d-amphetamine, and a DA-antagonist, haloperidol, respectively (Kumari et al., 1997). Further supporting a strong dopaminergic basis of PL, patients with schizophrenia given DRD2 blocking typical antipsychotics (e.g., Green et al., 1997; Kern et al., 1998; Kumari et al., 2002), but not atypical antipsychotics (Purdon et al., 2002, 2003; Kumari et al., 2008b), show significant PL impairment.

Neurally, the basal ganglia, in particular the striatum, and the cerebellum are known to play important roles in PL, based on the observations of impaired PL on variants of the SRT in patients with Parkinson's disease (Knowlton et al., 1996; Foerde and Shohamy, 2011), Huntington's disease (Heindel et al., 1989; Knopman and Nissen, 1991; Willingham et al., 1996) and damage to the cerebellum (Pascual-Leone et al., 1993; Molinari et al., 1997; Gomez-Beldarrain et al., 1998). With the striatum (Alexander and Crutcher, 1990) and cerebellum (Schmahmann, 1991) both projecting to the frontal lobe via the thalamus, the frontal cortex is also thought to be a component of the circuit subserving PL (Doyon et al., 1996; Honda et al., 1998; Gomez-Beldarrain et al., 1999). Neuroimaging evidence confirms the involvement of these regions in PL (Jenkins et al., 1994; Doyon et al., 1997; Kumari et al., 2002) and, in addition, shows involvement of the thalamus and cingulate gyrus (Kumari et al., 2002). Considering the various regions involved in PL, DA appears to be a key neurotransmitter given its prominence in the basal ganglia, frontal lobe and the thalamus which contains the highest levels of DRD2 receptors out of all extrastriatal brain regions (Kessler et al., 1993; Hall et al., 1996).

This is the first study, to our knowledge, to examine psychosis-proneness personality as well as the discriminant validity of schizotypy and psychoticism in an fMRI study of PL. Based on separate strands of previous evidence concerning the behavioral effect of DA-agonists and antagonists on PL (Kumari et al., 1997), and the imaging literature on PL, we hypothesized that psychosis-proneness personality, given its overlap with positive (hyper-dopaminergic) symptoms of psychosis, would correlate positively with PL and related brain activity, especially in dopamine-rich regions such as the striatum and the thalamus.

## Materials and methods

### Participants

Thirty healthy individuals (15 men, 15 women) were recruited from the general population using advertisements, flyers and mailing lists. All participants were right-handed and were screened for a history of substance and alcohol abuse, anorexia, mental illness, and regular medical prescriptions. A semi-structured interview was conducted to rule out the presence of a mental disorder and the presence of psychosis in their first-degree relatives. Of 30 individuals recruited initially, 26 individuals (13 men, 13 women) were included in the final sample. Of four individuals who were not included in the final sample, two did not fully complete the personality questionnaires, one had missing online performance data due to problems with the button box, and one provided unusable fMRI data.

The study procedures were approved by the Ethics Committee of the Institute of Psychiatry and the South London and Maudsley NHS Trust. All participants provided written informed consent after the study procedures had been fully explained to them.

### Psychometric assessment

A number of self-report rating scales can be used to assess psychosis-prone personality factors. In this study, the level of psychosis-proneness in each participant was assessed using two questionnaires: (1) the Psychoticism scale of the Eysenck Personality Questionnaire-Revised (EPQ-R; Eysenck and Eysenck, 1991) and (2) The Schizotypal Personality Scale (STA; Claridge and Broks, 1984). The EPQ-R P scale is proposed to be a general measure of the putative liability to the psychosis spectrum. The scale also includes items on antisocial, criminal and impulsive behaviors. The STA (Claridge and Broks, 1984) is a specific measure of schizotypal personality based on clinical observation. The scale focuses on positive schizotypy, including items on magical and delusional thinking as well as perceptual distortions. On each of the administered scales, higher scores indicate higher levels of self-reported psychosis-proneness. In addition to P, the EPQ-R measures two other major dimensions of personality, namely Extraversion (E) and Neuroticism (N), which were used in additional analyses, as described further.

### Procedure

Participants performed a 5-min sequence learning task in a blocked AB design, as described previously by Kumari and colleagues (2002, 2008b), while undergoing fMRI.

**Figure 1:**
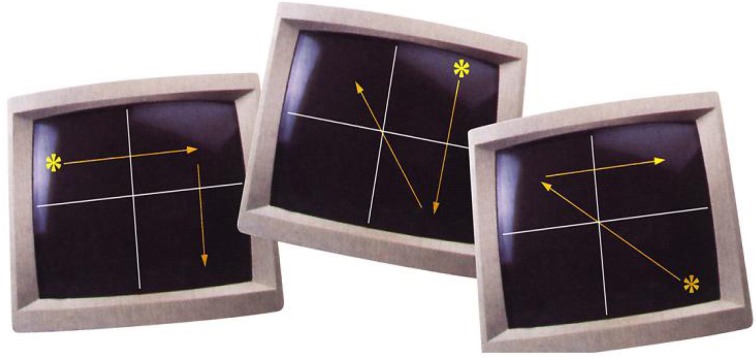
**Illustration of pattern trials (taken from Kumari et al., 2002)**.

The task consisted of two 30-s alternating conditions: blocks of random trials (OFF, control condition) and blocks of pattern trials (ON, experimental condition). In total, there were five blocks of random trials and five blocks of pattern trials. Participants were presented with a white target stimulus (an asterisk) on a black screen, viewed via a prismatic mirror fitted in the radiofrequency head coil, as they lay in the scanner. This target moved between four locations on the screen, which was divided into four equal quadrants by two intersecting white lines. The target movements during the pattern trials were predictable for 75% of cases, i.e., determined following three specific rules: (1) a horizontal target movement was followed by a vertical target movement; (2) a vertical target movement was followed by a diagonal target movement; (3) a diagonal target movement was followed by a horizontal movement. The fourth movement of the target during the pattern trials was unpredictable, which then was followed by the above mentioned three specific rules (Figure 1).

Participants were not told of the existence of specific rules governing the target movements during the pattern blocks, and the beginning of random and pattern blocks ware not marked in any way. They were asked to follow each target movement with their right hand as fast as possible using a MR compatible key pad with four keys, each key corresponding to one of the four quadrants. The movement of the target was initiated by the participants' touching the target key. Reaction times (RTs) were recorded on-line.

Prior to scanning, all participants underwent a practice session during which they practiced on five 30-s blocks of random trials and five 30-s blocks of pattern trials, both alternated with 30-s rest periods, in order to familiarize themselves with task requirements and the use of the MR compatible key pad. The practice session was identical for all participants.

### Image acquisition

Echoplanar MR brain images were acquired using a 1.5 T GE Signa system (General Electric, Milwaukee WI, US) at the Maudsley Hospital, London. Daily quality assurance was carried out to ensure high signal to ghost ratio, consistent high signal to noise ratio, and excellent temporal stability using an automated quality control procedure. A quadrature birdcage head coil was used for radio frequency (RF) transmission and reception. In each of 16 near-axial, non-contiguous planes parallel to the intercommissural (AC-PC) plane, 100 T2^*^-weighted MR images depicting blood oxygenation level-dependent (BOLD) contrast (Ogawa et al., 1990) were acquired over the 5-min experiment with echo time (TE) = 40 ms, repetition time (TR) = 3 s, in-plane resolution = 3.1 mm, slice thickness = 7.0 mm, and interslice gap = 0.77 mm. Head movement was limited by foam padding within the head coil and a restraining band across the forehead. At the same session, a high resolution 3-D inversion recovery prepared spoiled GRASS volume dataset was acquired in the AC-PC plane with TE = 5.3 ms, inversion time (TI) = 300 ms, TR = 12.2 s, in-plane resolution = 0.94 mm, and slice thickness = 1.5 mm.

### Data analysis

#### Behavioral data analysis

To examine the task effect (i.e., the presence of PL), mean RTs to blocks of random and pattern trials were subjected to a repeated measures analysis of variance (ANOVA) with Trial Type (random, pattern) and Block (1–5) as within-subjects factors. The amount of PL was calculated as the difference between the mean RTs to random and pattern trials. The possible association between the amount of PL and personality scores was examined with correlational analysis (Pearson's r). Statistical analyses were performed using SPSS for Windows (version 20.0). The alpha level of testing significance was kept at *p* = 0.05, unless stated otherwise.

#### Image processing

All images were processed and analyzed using Statistical Parametric Mapping software (SPM8; http://www.fil.ion.ucl.ac.uk/spm/). For each participant, the 100 volume functional time series was motion corrected (Friston et al., 1996), transformed into standard stereotactic Montreal Neurological Institute (MNI) space, spatially smoothed with an isotropic Gaussian kernel of 8 mm full width height maximum and band pass filtered (high-pass filter with cut-off at 128 s) using statistical parametric mapping software.

#### Models and statistical inferences

Functional MRI data were analyzed using a random effect procedure (Friston et al., 1999). This analysis consisted of a 30-s boxcar design (convolved with the haemodynamic response function) modeling the experimental condition (pattern trials). The control condition (random trials) formed the model's implicit baseline. Motion parameters were included as covariates at this stage. The second stage of the random effect model tested for generic activations across all participants' images using a one-sample *t*-test. The relationship of EPQ-R P scores with neural activity across the whole brain was identified using a multiple regression model (Psychoticism, Extraversion, Neuroticism, and PL scores entered into the model) within SPM8. In this analysis, the effects that survived *p* < 0.05, after correction for multiple comparisons at the cluster level (height threshold *p* < 0.01), were considered significant. A similar analysis strategy was used to examine possible association of STA schizotypy scores with BOLD signal in a separate model (i.e., a multiple regression model with STA schizotypy, EPQ-R Neuroticism and PL scores).

## Results

### Behavioral measures

The sample characteristics are described in Table 1. Inter-correlations between various personality and PL measures are presented in Table 2.

**Table 1:** **Sample characteristics**.

**Characteristic**	**Men**	**Women**	**All**
	**(*n* = 13)**	**(*n* = 13)**	**(*n* = 26)**
	**Mean (*SD*), range**	**Mean (*SD*), range**	**Mean (*SD*), range**
Age (years)	33.69 (11.92), 20–60	33.54 (14.89), 23–65	33.62 (13.21), 20–65
EPQ-R: extraversion	16.46 (4.27), 6–23	14.15 (4.18), 8–20	15.31 (4.31), 6–23
EPQ-R: neuroticism	7.46 (4.43), 1–16	10.31 (6.16), 0–20	8.88 (5.45), 0–20
EPQ-R: psychoticism	6.92 (3.84), 2–14	5.77 (3.47), 1–14	6.35 (3.63), 1–14
EPQ-R: lie	9.23 (4.25), 2–18	8.00 (3.87), 3–17	8.62 (4.03), 2–18
STA: schizotypy	7.46 (3.31), 1–13	5.08 (3.68), 0–12	6.23 (3.64), 0–13

*EPQ-R, Eysenck Personality Questionnaire-Revised; STA, Schizotypal Personality Scale*.

**Table 2:** **Inter-correlations (2-tailed) among personality measures and their relationship with PL scores**.

**Personality**	**EPQ-R: extraversion**	**EPQ-R: neuroticism**	**EPQ-R: psychoticism**	**STA: schizotypy**
	***r* (*p*)**	***r* (*p*)**	***r* (*p*)**	***r* (*p*)**
EPQ: neuroticism	−0.288 (0.153)			
EPQ: psychoticism	−0.130 (0.527)	0.133 (0.516)		
EPQ: lie	0.212 (0.298)	−0.089 (0.664)	−0.250 (0.218)	
STA: schizotypy	0.081 (0.693)	**0.471** (0.015)	**0.431** (0.028)	
Mean PL	0.253 (0.213)	−0.137 (0.506)	0.208 (0.307)	0.256 (0.208)

*EPQ-R, Eysenck Personality Questionnaire-Revised; STA, Schizotypal Personality Scale*.

**Figure 2:**
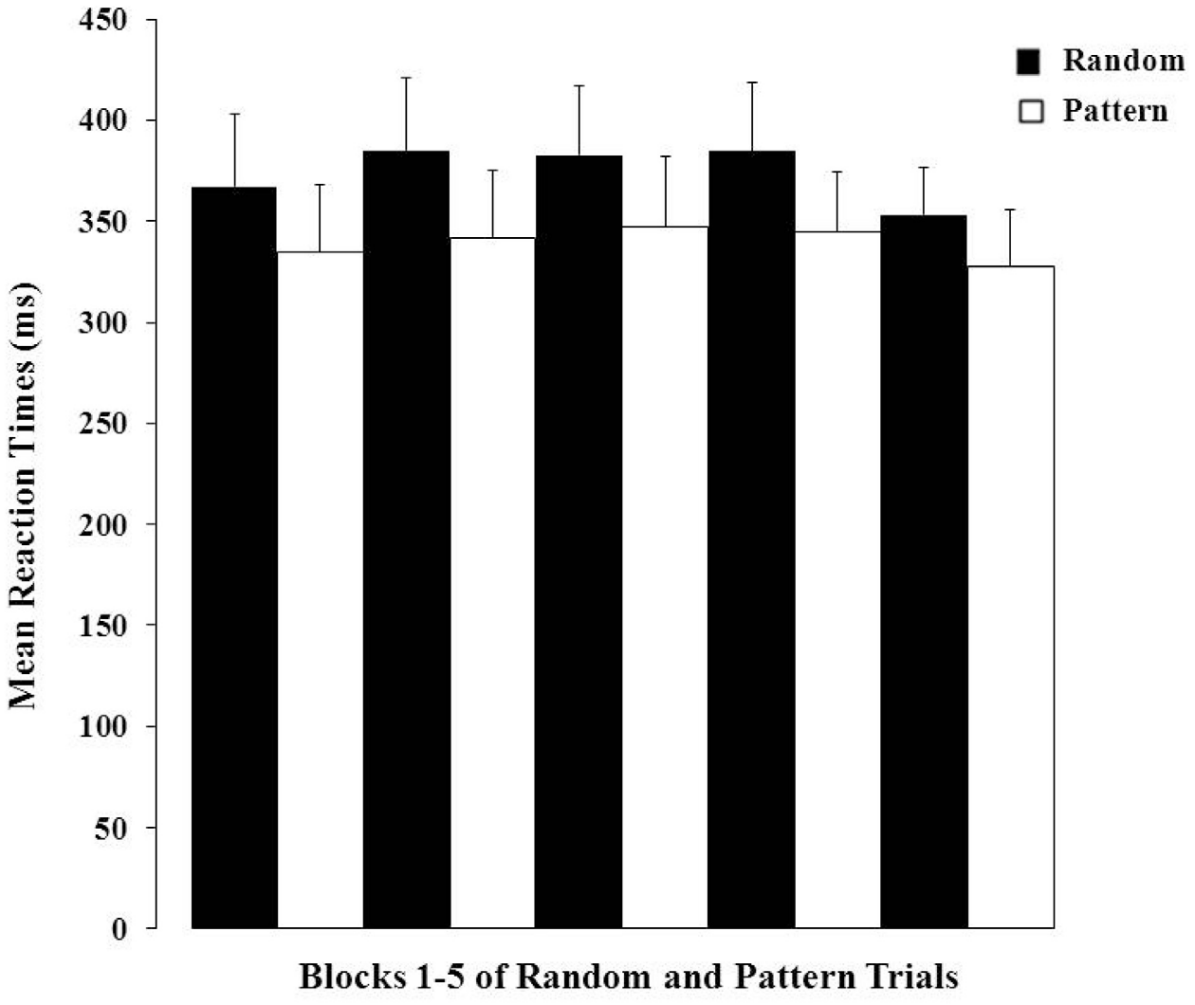
**Mean reaction times (+1 SEM) to random and pattern trials over the five blocks**.

There was a highly significant effect of Trial Type (*F* = 29.84, *df* = 1, 100, *p* < 0.001) demonstrating strong PL over the entire session (i.e., shorter RTs on pattern relative to random trials; Figure 2). The Block main effect and Trial Type × Block interaction effect were not significant (*p*-values > 0.20). The data (Figure 2) obtained during the first block (30-s OFF and 30-s ON) of trials suggest that our participants were able to gain from the practice session they had prior to entering the scanner as they showed evidence of learning in the very first block of trials.

**Figure 3:**
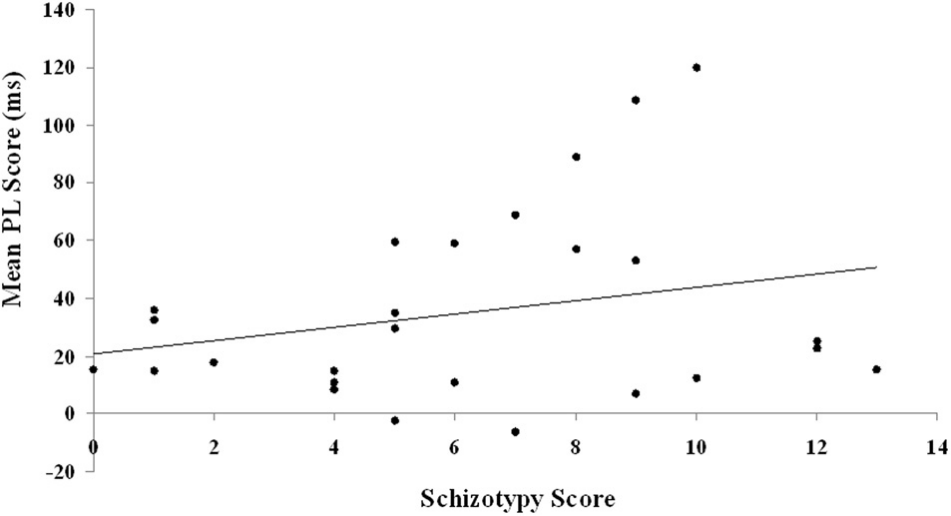
**Relationship between schizotypy and PL scores.** PL is calculated as the difference in reaction time between blocks of random trials and blocks of pattern trials. The schizotypy score are derived from the STA.

The two putative measures of psychosis-prone personality, the EPQ-R P and STA schizotypy scores, correlated significantly positively with each other. The STA schizotypy scores also correlated significantly positively with the EPQ-R Neuroticism scores (Table 2). None of the EPQ-R dimensions or STA schizotypy scores correlated significantly with PL scores. However, the relationship between STA schizotypy and PL became significant in the expected direction (i.e., positive) when we controlled for EPQ-R Neuroticism scores (partial correlation = 0.366, 1-tailed *p* = 0.036) (Figure 3).

### Functional MRI

#### Group activation

**Figure 4:**
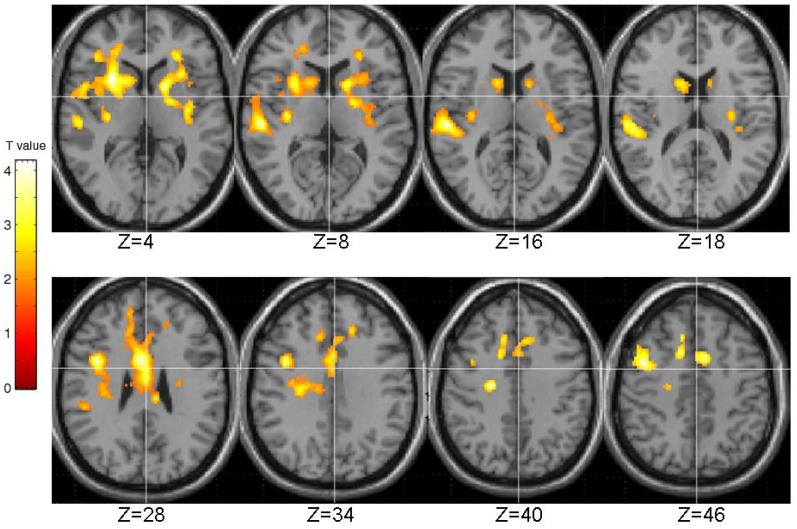
**Activation across all participants during pattern, relative to random, trials.** Images are left-right reversed.

The generic activation across all participants in association with PL is shown in Figure 4. Areas of stronger BOLD signal during PL than control blocks included a large cluster (number of contiguous voxels = 7558; FWE-corrected *p* = 0.001) with peak in the inferior frontal gyrus [BA9; (*x*, *y*, *x*) 40, 6, 30; voxel *T* = 4.19], subpeaks in the anterior cingulate (BA24; 4, 6, 28; *T* = 4.16), putamen (28, 16, 4; voxel *T* = 3.65 and −16, 8, 6; *T* = 3.34), middle frontal gyrus (BA6; 46, 6, 48; *T* = 3.39), and extending to the caudate (bilateral) and insula (left) (Figure 4).

#### Psychosis-proneness and brain activity

**Figure 5:**
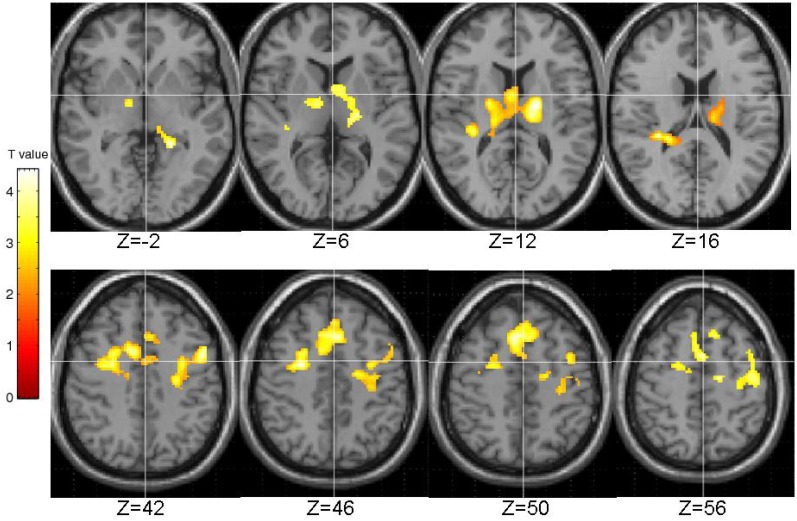
**Brain activity positively correlated with the EPQ-R Psychoticism (P) scores.** Images are left-right reversed.

The EPQ-R P scores correlated significantly positively with activity during PL in three clusters: (1) the right transverse temporal gyrus extending to the putamen, caudate, thalamus and insula; (2) the inferior frontal and precentral gyri; and (3) middle frontal gyrus extending to the precentral gyrus and anterior cingulate (Table 3, Figure 5).

**Table 3:** **Brain regions demonstrating positive associations with EPQ-R Psychoticism**.

**Brain region**	**BA**	**Cluster size**	**Side**	**MNI coordinates**			***T*-value**	**Cluster FWE-corrected *P***
				***x***	***y***	***z***		
Transverse temporal gyrus	41	1734	Right	32	−32	18	6.09	0.001
Insula	13		Right	24	−38	20	5.31	
Putamen	n/a		Right	18	−8	12	5.19	
Thalamus	n/a		Left	−14	−16	14	4.72	
Parahippocampal gyrus	30		Left	−20	−40	−2	4.40	
Transverse temporal gyrus	41		Right	36	−28	10	4.39	
Putamen	n/a		Right	22	−6	10	4.44	
Thalamus	n/a		Right	16	−22	10	3.96	
	n/a		Right	2	−2	14	3.88	
Caudate	n/a		Left	−8	6	2	3.15	
Inferior frontal gyrus	9	1322	Left	−48	6	40	5.07	0.007
Precentral gyrus	4		Left	−40	−18	60	4.59	
	4		Left	−26	−16	40	4.30	
Middle frontal gyrus	6	1730	Left	28	−4	46	4.77	0.001
Superior frontal gyrus	8		Left	8	20	50	4.74	
Anterior cingulate	32		Left	8	8	42	4.53	

*BA, Brodmann Area; MNI, Montreal Neurological Institute; FWE, Family Wise Error*.

**Figure 6:**
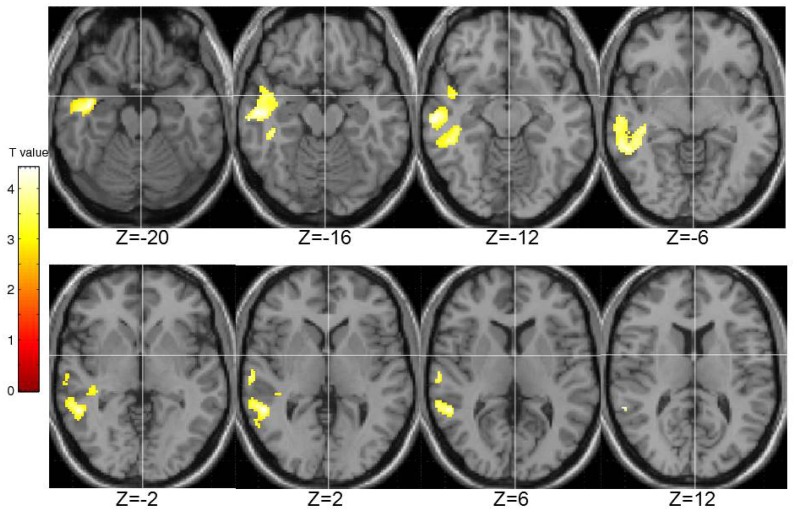
**Brain activity positively correlated with the STA schizotypy scores.** Images are left-right reversed.

STA schizotypy score correlated significantly positively with activity in only one cluster (number of contiguous voxels = 1450; FWE-corrected cluster *p* = 0.005) located in the right middle temporal gyrus (BA21; peak: 42, −6, −20; *T* = 4.36; sub-peaks: BA21; 54, −46, 2; *T* = 3.91; BA22; 62, −18, −12; *T* = 3.90). The extent of this cluster is displayed in Figure 6.

No brain area showed a significant negative correlation with EPQ-R P or STA schizotypy scores, and no area correlated significantly positively or negatively with the EPQ-R Extraversion or Neuroticism scores.

## Discussion

This study replicates the oft-repeated observation of PL in a motor sequence learning task (e.g., Corr et al., 1997). In comparison to blocks of trials where the target moved in a random pattern, there was a significant reduction (i.e., the main effect of Trial Type) in reaction time in blocks where the target moved in a repetitive, and thus predictable, sequence. This pattern suggests that the motor sequence was (implicitly) learnt and thus validates the paradigm for use in the current study in order to explore the association between brain function during PL and schizotypy. However, in this study as well as in healthy participants of our previous fMRI study with this paradigm (Kumari et al., 2002), there was evidence of learning in the very first block of trials, likely resulting from the practice session prior to fMRI experiment, and, as a result, no significant Block × Trial Type interaction. This study, therefore, is likely to have identified brain regions associated with recall, and not acquisition, of implicit knowledge about the sequences.

The pattern of brain activation that was observed during PL at the group level is mostly consistent with previous fMRI studies using this task (Kumari et al., 2002, 2008b). We observed increased BOLD signal during blocks involving patterned sequences than during blocks involving random sequences in the inferior frontal gyrus, anterior cingulate, middle frontal gyrus, insula, and striatum. The involvement of dopaminergic regions such as the striatum is to be expected given the previously described link between DA (a prominent neurotransmitter in the striatum), Parkinson's disease (a neurological condition involving loss of nigrostriatal dopaminergic neurons) and PL impairment (Foerde and Shohamy, 2011). As described in the introduction, there is also evidence of dopaminergic influences on PL from pharmacological studies in healthy individuals and patients with a diagnosis of schizophrenia (Green et al., 1997; Kumari et al., 1997, 2002; Kern et al., 1998).

At the behavioral level there were no significant first-order correlations between the amount of PL and different measures of psychosis-prone personality factors. However, when covarying for neuroticism, the correlation between positive schizotypy STA and PL became significant in the expected direction, indicating more PL in higher schizotypy. Neuroticism is known to be correlated with measures of schizotypy (e.g., Eysenck and Barrett, 1993; Lipp et al., 1994) and increased levels of neuroticism are observed in patients with a diagnosis of schizophrenia (Berenbaum and Fujita, 1994; Catts et al., 2000). A recent twin study showed that the overlap between neuroticism and positive schizotypy is largely of genetic origin (Macare et al., 2012).

Due to the observation of a relationship between schizotypy and neuroticism, a number of previous studies have investigated the relationship between schizotypy and cognitive performance whilst covarying for individual differences in neuroticism (Braunstein-Bercovitz, 2000; Ettinger et al., 2005; Völter et al., 2012). Significant partial correlations in those studies indicate an association of schizotypy with cognition over and above any contributions from neuroticism. Here we observed that the correlation between positive schizotypy STA and PL became significant only after including neuroticism as a covariate.

At the brain functional level there were significant positive correlations between psychosis-prone personality factors and BOLD signal in a number of areas. Higher EPQ-R P scores were associated with higher brain activity during PL in temporal cortex, striatum, thalamus, inferior frontal areas, middle frontal gyrus, and anterior cingulate. Higher STA scores on the other hand were associated with higher brain activity only in the right middle temporal gyrus.

On the basis of previous evidence of a relationship between DA and PL, we had expected that individual differences in PL would be associated primarily with differences in brain activity in dopaminergic regions in the striatum and thalamus. The current findings, at least in terms of individual differences as assessed by EPQ-R P scale, are in agreement with this expectation. Additionally, the same relationship was found between psychosis-prone personality factors and clusters in the frontal lobe. It should be emphasized that the direction of the relationship between psychosis-proneness and fronto-striatal-thalamic activity was *positive* in the present study but *negative* in our previous fMRI studies that involved tasks requiring involuntary (prepulse inhibition; Kumari et al., 2008a) or voluntary inhibition (antisaccade; Aichert et al., 2012). Taken together such results, although not surprising given that increased DA activity is known to disrupt performance on inhibitory tasks such as prepulse inhibition (Swerdlow et al., 2008) but to increase PL (Kumari et al., 1997), indicate that the direction of the association of psychosis-prone personality factors with fronto-striato-thalamic activity is situation specific (i.e., negative during inhibitory tasks; positive during automatic tasks facilitated by practice) rather than static. There is further support for this position from other studies (e.g., Szymura et al., 2007) showing that psychosis-proneness, as assessed with EPQ-R P, facilitates performance of simple tasks but leads to impairment on complex ones requiring flexibility and effortful control (review; Corr, 2010).

Our findings in relation to correlates of the STA schizotypy and EPQ-R P scales were not identical at either the behavioral or neural levels. Although the EPQ-R P and STA schizotypy scales had a modestly positive association (*r* = 0.431) with each other, only the STA schizotypy scale had a positive correlation with EPQ-R Neuroticism (*r* = 0.471). Only the EPQ-R P, and not the STA schizotypy, had an association with activity in the basal ganglia and thalamus (with or without the EPQ-R Neuroticism in the model). Given the pattern of effects we observed in this study, it seems sensible to conceptualize the EPQ-R P and STA schizotypy scales as measuring related but distinct constructs (Pickering, 2004; Corr, 2010).

Another aspect of the present study deserving discussion is that, unlike the findings of a recent fMRI study (Corlett and Fletcher, 2012) showing a correlation between non-clinical schizotypal experiences and aberrant frontal and striatal prediction error signal, consistent with the deficits found in early psychosis (Corlett et al., 2007), the behavioral/brain effects we observed in relation to psychosis-proneness in this study are not in line with what we found earlier in unmedicated first episode patients (Kumari et al., 2008b). Although in our previous study (Kumari et al., 2008b) we had found somewhat faster PL (i.e., greater PL in earlier blocks) in unmedicated first episode patients than the healthy group, this had resulted from longer RTs to random trials, rather than faster RTs to pattern trials. As we discussed previously (Kumari et al., 2008b), this might have reflected a conscious or unconscious search on the part of patients for, or imagining, “specific patterns” in the random trials condition driven by the presence of paranoia and other positive symptoms, as can be inferred from some of the neurobiological models of positive symptoms (e.g., Kapur, 2003; Corlett et al., 2010). The pattern of results we find in relation to psychosis-proneness in this study (further confirmed by absence of any correlation between reactions times to random trials and schizotypy measures; data not shown) is however consistent with what we observed in healthy people following acute administration of 5 mg d-amphetamine (Kumari et al., 1997), i.e., faster RTs to pattern trials. It is also worth pointing out that while the effects of DA-blocking antipsychotics in schizophrenia patients seem fairly consistent (resulting in poor PL), this is not the case for a relationship between symptoms of schizophrenia and PL (e.g., Exner et al., 2006; Reiss et al., 2006). Thus, whilst our data suggest a link between DA, given previous data showing strong sensitivity of SRT to dopaminergic manipulations, and schizotypy/broader “psychosis-proneness,” consistent with the DA hypothesis of schizophrenia (Gray et al., 1991; Carpenter and Koenig, 2008; Howes and Kapur, 2009; Howes et al., 2012), they cannot be viewed as supporting the continuum between schizotypy and psychosis at the symptom levels. The study did not directly address the continuum between psychosis-prone personality and psychosis at particular symptom level, e.g., delusional beliefs.

An important feature of this study was the investigation of specificity of the observed effects. As we had collected data not only on psychosis-prone personality factors (psychoticism and STA) but also on neuroticism and extraversion, we were able to explore whether those traits were associated with PL or brain function. No such correlations were found (but see above for the role of neuroticism in the relationship between STA and PL), suggesting some specificity of the current findings across different personality traits. Future work will be required to further probe specificity within the spectrum of clinical phenotypes and their subclinical expressions, for example given evidence of altered motor sequence learning in attention deficit/hyperactivity disorder (ADHD) (Adi-Japha et al., 2011; Prehn-Kristensen et al., 2011) and autism (Mostofsky et al., 2000).

Limitations of the study include the relatively modest sample size. Therefore, replication of the design in independent, larger samples will be important in order to validate the current findings. A further limitation concerns the fact that this study is unable to address the continuum between psychosis-proneness personality and schizophrenia at specific symptom/dimension level. We did not include a separate specific measure for negative schizotypy which is measured with scales such as the Physical Anhedonia or Social Anhedonia scales (Chapman et al., 1976) or the Introvertive Anhedonia scale from the Oxford Liverpool Inventory of Feelings and Experiences (Mason et al., 1995). Negative schizotypy shows distinct cognitive, affective, genetic, and neural correlates from positive schizotypy (Suhr and Spitznagel, 2001; Ettinger et al., 2005; Holahan and O'Driscoll, 2005; Lewandowski et al., 2006; Soliman et al., 2008; Macare et al., 2012). Accordingly, it would have been of interest to investigate whether negative and positive schizotypy also differentially relate to the brain functional response during PL. Finally, it should of course be mentioned that this study did not include direct examination of DA function in relation to psychosis-proneness personality factors.

In conclusion, the present study shows that the well replicated PL effect in a motor sequence learning task is associated with increased activation in frontal and striatal areas. Individual differences in psychosis-prone personality factors are found to relate both to the amount of PL (when neuroticism is considered as covariate) and the brain functional response in frontal, striatal and thalamic brain areas. These data are interpreted as being supportive of a dopaminergic involvement in psychosis-proneness, at least when measured using EPQ-R P scale.

### Conflict of interest statement

The authors declare that the research was conducted in the absence of any commercial or financial relationships that could be construed as a potential conflict of interest.
